# The Science and Art of Surgery

**Published:** 1854-07

**Authors:** 


ART V.—*The Science and Art of Surgery*, by JOHN ERICHSEN, Professor of Surgery in University College, and Surgeon to University College Hospital, London. 8vo. p. 908.

To the medical profession generally, but more particularly to students, we can recommend with honest pleasure the above system of surgery lately issued from the prolific press of Blanchard & Lea, and judiciously edited by John H. Brinton, M. D. It is, in our humble judgement, decidedly the best book of the kind in the English language. Strange that just such books are not oftener produced by public teachers of Surgery in this country and Great Britain. Indeed, it is a matter of great astonishment, but no less true than astonishing that of the many works on surgery, republished in this country within the last fifteen or twenty years as text-books for medical students, this is the only one, we venture to state, that even approximates to the fulfillment of the peculiar wants of young men just entering upon the study of this branch of the profession. In saying this, it is as far from our intention as our ability to throw discredit upon the various American and foreign surgical authors with whose writings the profession has been favored within the time mentioned, for many of their contributions are of much more intrinsic value to the progress of scientific surgery than the work before us. Take for example "Miller's Principles of Surgery," "Paget's Surgical Pathology," or "Gross's Urinary Organs," than which we challenge any one to point us to more pilosophical and truly valuable productions in Europe or America. But these are not text-books, properly so called, and do not therefore come within the range of our remarks. It is true that "Miller's Principles" was professedly written for the use of students, but we take the liberty of assigning it a higher rank in surgical literature than that of a strictly elementary treatise. In regard to many other modern surgical authors who have attempted the production of works having the same object in view as that attained by Mr. Erichsen, and who instead of reaching a somewhat higher position as did Mr. Miller, have fallen far short of their aim, we have nothing here to say. Our wish is not to draw comparisons, but simply to express a favorable opinion of the book before us, and to point out some of its specially valuable parts and a few comparatively unimportant statements to which we make exceptions.

It will be seen by reference to the heading that the book although consisting of but one volume is no mere hand-book, but a voluminous tome of over nine hundred closely printed pages, illustrated by three hundred wood-cuts, executed in the highest style of the art, many of them entirely original and well adapted for all the purposes intended by such aids. Indeed we may say that no book of the same size on surgery with which we are acquainted contains so many good and well selected wood-cuts as the one before us.

As a matter of course—the habit being one acknowledged by all judicious teachers to be absolutely necessary—the first subject treated of is *Inflammation.* To this, and its immediate effects, namely, effusions of serum and lymph, suppuration, granulation, cicatrization and mortification, the author devotes about forty pages. Assuming, as almost every one will allow, that the proper estimate of the ability of a teacher of elementary surgery may be generally formed from his views in regard to and his presentation of the facts concerning this great fundamental action in the production and cure of disease, we think that the article in question almost fully sustains our previously expressed opinion of the book in general. We must say, however, that notwithstanding the author's great powers of condensation and generalization, we think he might have written eighty or a hundred pages on this subject without injustice to himself or his readers. In regard to his manner of treating the subject we see nothing peculiar or worthy of more than a general commendation. We beg leave however, in passing, to enter our *caveat* against the phraseology "secondary forms of inflammation" as applied to effusions of serum and lymph, suppuration, &c. These should be, and are by all intelligent pathologists considered as only *effects*, indicating, however, different stages or degrees of inflammatory action. Thus the first effect of developed inflammation in almost all the tissues of the body is an effusion of serum, which may be looked upon as marking the second stage or degree of the action, congestion being the first. A little higher degree is attended with the formation of lymph, the second effect or third stage; and so on in reference to suppuration and mortification, the last mentioned indicating the highest possible degree of action of which a tissue is susceptible. Ulceration, granulation, and cicatrization, although often very much to be desired, are only relative effects occurring under certain circumstances, such as the accident of situation, tissue, &c., and are not properly forms or degrees of inflammation, or, if looked upon in this light, should be placed upon the same level with suppuration. It is true that surgeons constantly make use of such expressions as "adhesive inflammation," "ulcerative inflammation," "gangrenous inflammation," &c., but we do not recollect of any one before denominating the degrees of action which they distinguish, as so many *scecondary forms* of inflammation.

Immediately succeeding the article on inflammation is one entitled *General Considerations on Operations*, embracing all the different kinds of amputations and occupying about thirty pages. The introduction of this subject here is, we think, decidedly ill judged, and we can only account for it by supposing the author to have been influenced mainly by a desire to depart from the old established routine of treating of wounds in this situation, and somewhat probably by the fact that there is no special connection between amputations and any single class of surgical diseases. Whatever may have been his reasons, we do not agree with him. There is such an evident connection between the subject of wounds and bruises in general and inflammation, that we see no necessity for parting them by interposing an account of operations, which it will be plenty soon enough for the student to learn when he has mastered nearly every thing else in surgery. The subject-matter of the article, however, is excellent, the descriptions clear and accurate, and the illustrations are all that can be desired in this way. We extract some instructive tables showing the comparative results: 1st, of amputations for injury and disease; 2nd, of primary and secondary amputations; 3d, the greater success obtained at the University Hospital, London, than that reported by Malgaigne of Paris. No one, we think, can read these tables without being convinced of the great superiority of English over French surgery.

RESULT OF AMPUTATIONS IN UNIVERSITY COLLEGE HOSPITAL.

*No. 1.—Injury.*

| Seat of Amputation. | No. of cases. | Cured. | Died. | Mortality per cent. |
|---|---|---|---|---|
| Thigh, | 19 | 8 | 11 | 58 |
| Leg and ankle, | 14 | 12 | 2 | 14 |
| Arm and shoulder, | 6 | 5 | 1 | 16½ |
| Fore-arm, | 6 | 6 | 0 | 00 |
| Total, | 45 | 31 | 14 | 31 |

*No. 2.—Disease.*

| Seat of Amputation. | No. of cases. | Cured. | Died. | Mortality per cent. |
|---|---|---|---|---|
| Thigh, | 34 | 27 | 7 | 20½ |
| Leg and ankle, | 39 | 32 | 7 | 18 |
| Arm and shoulder, | 13 | 9 | 4 | 30¾ |
| Fore-arm, | 9 | 9 | 0 | 00 |
| Total, | 95 | 77 | 18 | 19 |

---

MALGAIGNE'S STATISTICS.

*Amputations for Injury.*

| Seat of Amputation. | Cases. | Died. | Mortality per cent. |
|---|---|---|---|
| Thigh, | 46 | 34 | 75 |
| Leg, | 79 | 50 | 62 |
| Foot, | 9 | 6 | 66 |
| Arm, | 30 | 17 | 52 |

*Amputations for Disease.*

| Seat of Amputation. | Cases. | Died. | Mortality per cent. |
|---|---|---|---|
| Thigh, | 153 | 92 | 60 |
| Leg, | 112 | 55 | 50 |
| Foot, | 29 | 3 | 11 |
| Arm, | 61 | 24 | 40 |

*Primary Amputations.*

| | Cases. | Deaths. | |
|---|---|---|---|
| Thigh, | 6 | 3 | University College. |
| | 13 | 8 | James. |
| | 44 | 34 | Malgaigne. |
| | 5 | 5 | South. |
| | 11 | 11 | Laurie. |
| | 32 | 21 | Steele. |
| Leg, | 8 | 2 | University College. |
| | 18 | 7 | James. |
| | 67 | 42 | Malgaigne. |
| | 9 | 2 | South, |
| | 22 | 15 | Laurie. |
| | 53 | 22 | Steele. |
| Shoulder & arm, | 4 | 1 | University College. |
| | 19 | 3 | James. |
| | 36 | 24 | Malgaigne. |
| | 6 | 0 | South. |
| | 26 | 13 | Laurie. |
| | 49 | 15 | Steele. |
| Fore-arm, | 5 | 0 | University College. |
| | 18 | 0 | James. |
| | 10 | 2 | Malgaigne. |
| | 1 | 0 | South. |
| | 15 | 0 | Laurie. |
| | 35 | 1 | Steele. |

*Secondary Amputations.*

| | Cases. | Deaths. | |
|---|---|---|---|
| Thigh, | 13 | 8* | University College. |
| | 15 | 5 | James. |
| | 18 | 15 | Steele. |
| | 24 | 16 | Laurie. |
| Leg, | 6 | 0 | University College. |
| | 5 | 1 | James. |
| | 19 | 13 | Steele. |
| | 5 | 3 | Laurie. |
| Shoulder & arm, | 2 | 0 | University College. |
| | 4 | 1 | James. |
| | 16 | 9 | Steele. |
| | 14 | 7 | Laurie. |

* It is remarkable that at the University College Hospital, secondary amputations of the thigh have been more fatal than the primary ; but the number of cases (nineteen in all) is too small to vitiate the general law.

The second division of the work, embracing over two hundred pages, is devoted to *Surgical Injuries*, comprising wounds of the soft parts, fractures, dislocations, asphyxia, foreign bodies in the air-passages, burns, and frost-bites.

Under the head of wounds of the soft parts, the author makes the usual divisions into incised, bruised, lacerated, gun-shot, &c., points out in a very systematic and decided manner the indications to be fulfilled, and advocates strongly simplicity of local treatment and a strict attention to general condition. The article is one every way worthy of a sound and judicious practitioner and an able instructor, excepting probably a little disposition to over-refinement in theory. We are happy to observe, however, that his practice is sound, although inconsistent with some of the refined doctrines which he sets forth. Thus in speaking of incised wounds, he mentions *five* entirely different modes by which union may be effected—more than twice the number admitted by any author or teacher of surgery previous to the late McCARTNEY, of Dublin. The five processes described by Mr. ERICHSEN are denominated:—1st. direct growing together of opposed surfaces; 2nd. scabbing; 3d. union of opposite surfaces through the medium of coagulable lymph (union by first intention of the old surgeons); 4th. by granulations springing up from the sides and bottom and thus filling up the wound; and 5th. by the growing together of two granulating surfaces.

It will thus be seen that our author admits two modes of union antecedent to, and of course, more to be desired than the old first intention or adhesive inflammation. The first mentioned, namely the immediate growing together of newly cut surfaces without effusion of lymph, was first described by the celebrated Dublin surgeon just mentioned, who is said to have observed it in clean cut wounds of the fingers. It is also adopted by MILLER, and is said also to have received confirmation by observations of PAGET. The second mode, scabbing, is entirely new to us, although not original with our author, as it is also hinted at by MILLER. We give his own description of it.

"*Healing by scabbing* consists in the direct adhesion of the lower part and sides of a wound under a crust of dried blood, hair, &c., which forms an air-tight covering. The absence of inflammation is necessary for healing by scabbing; if inflammatory effusion occur, the scab will be thrown off, and air getting admitted an ulcer forms upon which granulations will spring up. This kind of union is extremely rare in man, owing to the readiness with which inflammation is excited, but is common in the lower animals, inflammation not being so readily induced, in them. This natural process is sometimes imitated by the surgeon closing a wound with a piece of lint steeped in blood or collodion under which union takes place."

We must confess our disbelief in any such process in man, and we very much doubt whether any well educated modern surgeon ever employed the mode of closing a wound, mentioned above, having the object in view stated by Mr. ERICHSEN. When union takes place under these circumstances it is undoubtedly by adhesive inflammation, the lymph of the effused blood probably playing an important part in the process as supposed by JOHN HUNTER. The only example of healing analogous to this scabbing process, of which we are aware, occurs after superficial burns and scalds, in which the true skin is not destroyed in its entire thickness, and if properly protected from the air gets covered by a new layer of epidermis by a simple exudation of nucleated blastema, whereas if exposed to the air takes on an inflammatory action and presents all the appearance of a granulating sore.

The inconsistency of the author's doctrines and practice may be seen by comparing this account of the healing processes with the subjoined directions for the management of incised wounds.

"In the treatment of an incised wound we must always endeavor to procure direct union, or adhesive inflammation, between a portion if not the whole of the surfaces." The question naturally suggests itself, if there be two modes of union superior to adhesion why not recommend some way to take advantage of them?

In turning over to the article on wounds of the abdomen, we find that our author gives to GUTHRIE and TRAVERS the whole credit of having settled the question in regard to the propriety of sewing up wounds of the intestines. He does not seem to be aware of the experiments of Prof. GROSS upon this same point, the results of which were published ten or twelve years ago in a monograph entitled "Wounds of the Intestines." This gentleman has done more, in our opinion, towards settling this question conclusively than either of the two mentioned. We are also somewhat astonished to find that Mr. Erichsen is not impressed with the abso lute necessity of taking deep stitches in sewing up wounds of the abdominal walls, involving their whole thickness. He simply says: "if the wound have not implicated any of the abdominal viscera, it must be closed by relaxing the abdominal muscles, by position, by introducing a few points of interrupted suture *through the integuments*, if it is extensive, and by applying a compress and plaster supported by a bandage." We hold that no experienced surgeon would trust to anything less than *several* interrupted sutures carried *down through the muscular substance* to within a short distance of the peritoneum, in a case of this sort. The failure to do this, has, in more instances than one, compromised the life of the patient, by allowing a protrusion of the intestines between the muscular layers as the writer had an opportunity of witnessing not many months since in a case occurring in the hands of a practitioner of this city.

Under the head of fractures, we are surprised and gratified at the simple yet satisfactory means recommended by the author. It seems that at the University College Hospital, of which the author is surgeon, special apparatus is almost entirely discarded in treating fractures of the extremities, and the starch bandage employed in nearly every case. We cannot resist the temptation of copying his remarks entire upon this point and recommend them to the careful perusal of all our readers who are interested in such matters:

"*Special apparatus* should be employed as little as possible in the treatment of fractures. It is scarcely ever necessary in simple fractures, and is far more cumbersome and costly than the means above indicated, which are all that can be required. I have no hesitation in saying that a surgeon of ordinary ingenuity and mechanical skill may be fully prepared to treat successfully every fracture to which he can be called, by having at hand a smooth deal plank half an inch in thickness, and a sheet of gutta percha, undressed sole leather, or pasteboard, to cut into splints as required.

"To these simple means the *starch bandage* is an invaluable addition. Although various plans for stiffening and fixing the bandages in cases of fractures, by smearing them with white of eggs, with gum, &c., have been employed at various times, it is only of late years that the full value of the starch bandage has been recognized by surgeons, chiefly through the practice and writings of M. SEUTIN of Brussels.

"The advantages of the starch bandage in the treatment of fractures, as well as in many other injuries and diseases, consist in its taking the shape of the limb accurately and readily, and maintaining it by its solidity; in being light, inexpensive, and easily applied, with materials always at hand. From its lightness it possesses the great and peculiar advantage, in fractures of the lower extremity, of allowing the patient to remain up, and to move about on crutches during nearly the whole of the treatment, and thus by rendering confinement to bed unnecessary, preventing the tendency to those injurious consequences that often result from these injuries; and by enabling the patient to keep up his health and strength by open air exercise, facilitating the consolidation of the fracture. In addition to this, the patient will often be able to carry on his business during treatment. By employing the starch bandage in the way that will be immediately pointed out, I scarcely ever find it necessary to keep patients with simple fractures of the leg in bed for more than three or four days, thus saving much of the tediousness and danger of the treatment.

"The following is the mode of applying this apparatus that is adopted at the University College Hospital, and that will always be found to answer: A dry roller is first applied to the limb, the osseous prominences of which are carefully and thickly padded with cotton wadding. If it be the lower extremity that is injured, and the fracture is very movable, a many tailed bandage will be found to be most convenient. This must be smeared with stiff starch; over this should be laid splints of thick and coarse paste-board, properly cut to fit the limb, extending beyond the joints nearest the fracture, and well soaked in thin starch. If much strength is not required, as in children, or in some fractures of the upper extremity, a few slips of brown paper well starched may be substituted for the paste-board. A bandage saturated with thick starch must now be applied; and, lastly, this is to be covered by another dry roller, the inner sides of the turns of which may be starched as it is laid on. During the application of this apparatus extension must be kept up by an assistant, so as to keep the fracture in position; and until the starch has thoroughly dried, which usually takes from thirty to fifty hours, a temporary wooden splint may be applied to the limb, so as to keep it to its proper length and shape. The drying of the starch may, if necessary, be hastened by the application of hot sand bags to the apparatus. After the bandages have become quite dry the temporary splints must be removed, and the patient may then be allowed to move about on crutches, taking care, of course, to keep the injured limb well swung up, and not to bear upon it or to jar it against the ground. In the course of about three or four days after its application, the apparatus will usually be found to have loosened somewhat, the limb appearing to shrink within it. Under these circumstances it becomes necessary to cut it up with a pair of Seutin's pliers. This section must be made along the more muscular part of the limb, so that the skin covering the bones be not injured, and after paring the edges of the splints it must be re-applied by means of tapes or a roller. In some cases it will be found advantageous to adopt the practice of M. Burggræve of Gent, and to envelop the whole limb in a thick layer of cotton wadding before applying the starched bandage; this being elastic accommodates itself to the diminution in the size of the limb, and thus keeps up more equable pressure. Indeed, of late, I have invariably adopted this practice, and found much advantage from it. In trimming the edges of the splint it should not be removed from the limb, and after this has been done, the apparatus must be tied together again with tapes or a roller. If the fracture be compound a trapdoor may be cut in the apparatus opposite the seat of the injury, through which the wound may be dressed.

Although fully recognizing the great advantage to be obtained by treating fractures on this plan, and employing the starch bandage in almost every case that came under my call, I did not think that it was a safe practice to have recourse to it during the early stages of fracture until, indeed, the swelling of the limb had begun to subside. I therefore never applied it until the sixth or eighth or tenth day, keeping the limb properly reduced upon a splint, very lightly bandaged, wet with cold evaporating lotions until this time, fearing that if the bandage were applied at too early a period, the inflammatory turgescence of the limb might give rise to a slow strangulation of it under the bandages. During the last summer, however, I have followed Seutin's plan in a great number of cases, at least fifty or sixty, of fractures of all kinds, putting the limb up in the starch apparatus *immediately* on the occurrence of the injury, and have found the practice an exceedingly successful one, even in fractures of the thigh; so much so that at the hospital I now rarely use any other plan of treatment. I find that the moderate pressure of the bandages aided probably by the great evaporation that goes on during the drying of so extensive and thick a mass of wet starch, and which produces distinct sensations of cold in the limb, takes down the extravasation most effectually, and enables the patient usually to leave his bed about the third day after the injury when the fracture is in the leg or ankle, and about the sixth when it is the thigh that is broken; so that very commonly we now treat all simple fractures of the leg and many of those of the thigh, especially in children, as out-patients."

Space will not allow us to go father into Mr. Erichsen's book at present, but we may refer to it again in some future number of our Journal, and in a way we hope that will prove more satisfactory to the reader than this brief introduction. In the mean time, we must thank the publishers for the handsome manner in which the volume is gotten up, and hope, not so much for their sake as for that of its readers, that it will meet with a most abundant sale.

